# Design of Potent, Non-Toxic Antimicrobial Agents Derived from the Frog Skin Peptide Raniseptin-PL Active Against Gram-Negative ESKAPE^+^ Pathogens

**DOI:** 10.3390/antibiotics15100944

**Published:** 2026-09-23

**Authors:** J. Michael Conlon, Milena Mechkarska, Taylor S. Cunning, Ryan Connolly, Nigel G. Ternan, Laure Guilhaudis

**Affiliations:** 1 Diabetes Research Centre, School of Biomedical Sciences, Ulster University, Coleraine BT52 1SA, UK; 2 Department of Life Sciences, Faculty of Science and Technology, University of the West Indies, St Augustine Campus, St. Augustine, Trinidad and Tobago; milena.mechkarska@sta.uwi.edu; 3 Nutrition Innovation Centre for Food and Health (NICHE), School of Biomedical Sciences, Ulster University, Coleraine BT52 1SA, UK; t.cunning@ulster.ac.uk (T.S.C.); connolly-r18@ulster.ac.uk (R.C.); ng.ternan@ulster.ac.uk (N.G.T.); 4 Université Rouen Normandie, INSA Rouen Normandie, Université Caen Normandie, ENSICAEN, CNRS, Institut CARMeN UMR 6064, F-76000 Rouen, France; laure.guilhaudis@univ-rouen.fr

**Keywords:** frog skin, raniseptin-PL, antimicrobial peptide, ESKAPE^+^, cytotoxicity, circular dichroism

## Abstract

**Background/Objectives**: The frog skin peptide raniseptin-PL (GVFDTVKKIGKAVGKFALGVAKNYLNS.NH_2_) is active against a range of antibiotic-resistant bacteria including ESKAPE^+^ pathogens, but its therapeutic potential is limited by high cytotoxicity against mammalian cells. The study aimed to determine the effects of selective amino acid substitutions by L- and D-lysine residues on the biological activity of the peptide. **Methods**: Conformational analysis of raniseptin-PL and its analogs was accomplished using circular dichroism spectroscopy. Antimicrobial activity was determined using reference strains and antibiotic-resistant strains of ESKAPE^+^ bacteria, and cytotoxicity against human neoplastic and non-neoplastic cells was evaluated. **Results**: Raniseptin-PL adopts an extended helical conformation in membrane-mimetic environments. Replacement of the Asp-4 residue by either L-Lys or D-Lys or the Leu-18 residue by L-Lys produced only modest changes in antimicrobial potency but high cytotoxicity against human-derived cells was retained. In contrast, substitution at Leu-18 by D-Lys attenuated potency against the Gram-positive bacteria *Staphylococcus aureus* and *Staphylococcus epidermidis* (MIC ≥ 50 µg.mL^−1^), but high potency against the antibiotic-resistant Gram-negative ESKAPE^+^ pathogens *Acinetobacter baumannii*, *Klebsiella pneumoniae*, *Pseudomonas aeruginosa* and *Escherichia coli* was either maintained or increased (MIC 3.1–12.5 µg.mL^−1^; approx.1–4 µM). Importantly, [L18k]raniseptin-PL and [D4k,L18k]raniseptin-PL lacked cytotoxic activity against human-derived HaCaT keratinocytes, A549 non-small cell lung adenocarcinoma cells, PC-3 prostate cancer cells and erythrocytes at concentrations up to 100 µM. **Conclusions**: [L18k]raniseptin-PL and [D4k,L18k]raniseptin-PL show therapeutic potential as templates for development of a non-toxic antimicrobial agent for use against systemic infections by Gram-negative bacteria, particularly ESKAPE^+^ pathogens.

## 1. Introduction

The emergence of strains of pathogenic bacteria with resistance to commonly used antibiotics constitutes an ever-increasing threat to public health [1]. Of particular concern is the widespread incidence, particularly in a hospital environment, of resistant ESKAPE pathogens [2,3]. The term ESKAPE refers to six virulent bacteria with a pronounced ability to develop resistance to commonly used antibiotics. The ESKAPE pathogens constitute the Gram-positive *Staphylococcus aureus* and *Enterococcus faecium* and the Gram-negative *Acinetobacter baumannii*, *Enterobacter* spp., *Klebsiella pneumoniae*, and *Pseudomonas aeruginosa* and are the leading cause of nosocomial infections worldwide [4,5]. When the multi-drug-resistant Gram-negative *Escherichia coli* is included in the group, the terms ESKAPE^+^ or ESKAPEE are sometimes used. Treatment of infections caused by ESKAPE^+^ pathogens represents a serious challenge to the physician and such infections are often associated with poor clinical outcomes and high mortality, particularly in immunocompromised patients [5,6]. Consequently, it is a matter of some urgency to develop potent, non-toxic agents to which these microorganisms have not developed resistance.

Naturally occurring peptides present in animal venoms and defensive secretions have been extensively investigated as potential antimicrobial agents for use against antibiotic-resistant microorganisms [7]. Of particular relevance to the present investigation is the observation, that skin secretions produced by some, but by no means all, species of Anura (frogs and toads) contain host-defense peptides (HDPs) that possess the ability to permeabilize both the cell membrane of prokaryotes and the plasma membrane of mammalian cells leading to cell death [8,9,10]. Skin secretions from species belonging to the extensive genera Hylidae, Pipidae, and Ranidae are a particularly rich source of such antimicrobial peptides (reviewed in [11,12,13]). The vast majority of frog skin HDPs are cationic due to a preponderance of lysine, and less commonly arginine, residues and have the propensity to adopt an amphipathic α-helix in membrane-mimetic environments such as trifluoroethanol (TFE)–water mixtures or in the presence of zwitterionic micelles formed from dodecylphosphocholine (DPC) or anionic micelles formed from sodium dodecyl sulfate (SDS) [14].

A previous study identified a range of peptides with potent broad-spectrum activity against bacteria and human tumor-derived cells in norepinephrine-stimulated skin secretions from the Caribbean banana tree dwelling frog *Boana platanera* (Hylidae) [15]. One such HDP, raniseptin-PL (GVFDTVKKIGKAVGKFALGVAKNYLNS.NH_2_), displayed rapid and potent activity against a range of Gram-positive and Gram-negative aerobic bacteria including ESKAPE^+^ pathogens and against the anaerobe *Clostridioides difficile*. However, the therapeutic potential of raniseptin-PL was severely limited by the fact that the peptide showed potent in vitro cytotoxic activity against a range of neoplastic and non-neoplastic human-derived cell lines. The aim of the present study was to design analogs of raniseptin-PL with substantially decreased cytotoxicity that retain potent antimicrobial activity for use as a template for the development of a therapeutically valuable non-toxic agent with anti-infective properties, particularly for use against ESKAPE^+^ pathogens.

## 2. Results

### 2.1. Conformational Analysis

The physicochemical properties (molecular charge at pH 7.0, hydrophobicity and hydrophobic moment (a measure of the degree of amphipathicity)) of raniseptin-PL and its analogs are compared in Table 1. Predictions obtained using PEP-FOLD4 (an ab initio approach specifically designed for peptides [16]) and the AlphaFold server (deep learning-based modeling [17]) indicate that the peptide has a strong propensity to adopt an α-helical conformation in residues 3–26 (PEP-FOLD4) or residues 4–24 (AlphaFold). A helical wheel representation of the peptide illustrates the amphipathic nature of the helix with the hydrophilic residues Asp^4^, Lys^8^, Lys^11^, and Lys^15^ segregating on one face of the helix and strongly hydrophobic residues on the opposite face (Figure 1).

Substitution of the Asp^4^ residue by Lys increases the molecular charge from +5 to +7 at pH 7.0 with only minor changes in hydrophobicity or hydrophobic moment. In contrast, substitution of Leu^18^ by Lys increases the molecular charge from +5 to +6 and, by extending the hydrophilic face of the helix, increases appreciably the hydrophobic moment. The polar angle θ (the angle subtended by the positively charged amino acids) is increased from 80° to 120°.

As shown in Figure 2, CD spectra of raniseptin-PL confirm the prediction that the peptide adopts an extended helical conformation in a membrane-mimetic environment. In common with other frog skin HDPs, the peptide adopts a predominantly random coil confirmation in water but in 50% (*v*/*v*) TFE, 20 mM DPC and 20 mM SDS, the spectra display two characteristic negative minima at approximately 208 nm and 222 nm, and a positive maximum near 191 nm, indicative of a predominantly α-helical structure. The % helicity in these solvents calculated using the SELCON3 program ranged from 59% to 70%, corresponding to between 16 and 19 amino acids in a helical conformation. The % helicity calculated from the mean residue ellipticity at 222 nm ranged from 50% to 62% (Table 2). The high value for [θ]_222_/[θ]_208_ is consistent with a well-formed α-helix in membrane-mimetic environments.

For analogs in which a L-Lys residue replaces either Asp^4^ or Leu^18^, the intensity of the maximum near 191 nm in the CD spectra was significantly greater in micellar media, indicating an enhanced degree of structural organization. (Figure 3). Substitution of the Asp^4^ residue increased the calculated helical content from 70% in the native peptide to 74% in the presence of zwitterionic DPC micelles and to 77% in the presence of anionic SDS micelles (Table 2). Similarly, the substitution of Leu^18^ by Lys increased the helical content to 75% in a solution containing DPC micelles and to 75% in a solution containing SDS micelles (Table 2).

These experimental observations were further examined in light of the structural predictions obtained using PEP-FOLD4 and AlphaFold servers. PEP-FOLD4 predicted that raniseptin-PL adopts a predominantly α-helical conformation extending from approximately residue 3 to residue 27, with a region of lower helical propensity between residues 12 and 18 that, in some models, interrupted the helix into two distinct helical segments (Supplementary Figures S1 and S2). Both the L-Lys^4^ and L-Lys^18^ substitutions increased the predicted helical propensity within this central region, suggesting stabilization of the α-helical conformation. This prediction is in agreement with the increased helical content estimated from the CD spectra. AlphaFold predictions, however, revealed no marked differences in residue-specific α-helical propensity among the three peptides, with a highly conserved α-helical region extending approximately from residue 4 to residue 23 (Supplementary Figure S3). This is consistent with the similar [θ]_222_/[θ]_208_ ellipticity ratios measured in micellar environments, which ranged only from 0.86 to 0.89 (Table 2). Thus, despite the moderate increase in calculated α-helical content following Lys substitution, the overall spectroscopic characteristics of the α-helical conformation remained similar.

### 2.2. Antibacterial Activities

Raniseptin-PL displayed relatively potent bactericidal activity against reference strains of the Gram-negative ESKAPE^+^ pathogens *A. baumannii*, *E. coli*, and *P. aeruginosa*, against vancomycin-resistant *E. faecalis* and against the reference strain ATCC 25923 and the methicillin-resistant strain ATCC 43300 of Gram-positive *S. aureus* (MIC and MBC between 6.25 and 12.5 µg.mL^−1^; 2–4 µM) (Table 3). The peptide was less potently active against carbapenem-resistant *K. pneumoniae* and carbapenem-resistant *P. aeruginosa* (MIC 25–50 µg.mL^−1^). Replacement of the Asp^4^ residue by L-Lys produced no or only minor increases in antimicrobial activity except in the case of antibiotic-resistant *K. pneumoniae* (4-fold increase in potency) and antibiotic-resistant *P. aeruginosa* (8-fold increase in potency). Replacement of Leu^18^ by L-Lys resulted in 2-fold greater activity (MIC in the range 3.13–25 µg.mL^−1^) against the ESKAPE^+^ pathogens *E. coli*, *K. pneumoniae* and *P. aeruginosa* compared with raniseptin-PL but activity against the reference strain and the methicillin-resistant *S. aureus* strain was severely attenuated (MIC 50–100 µg.mL^−1^).

A differential effect on antimicrobial potency was observed when Leu^18^ was substituted by D-lysine. The potency of [L18k]raniseptin-PL and [D4k,L18k]raniseptin-PL against Gram-negative bacteria was either unchanged (*A. baumannii*) or increased 2- to 4-fold (*E. coli*, *E. faecalis*, *K. pneumoniae*, *P. aeruginosa*). In contrast, activity against Gram-positive *S. aureus* was abolished (MIC > 100 µg.mL^−1^) and the potency against *S. epidermidis* was attenuated 8- to 16-fold.

### 2.3. Cytotoxic Activities

The effects of increasing concentrations of raniseptin-PL and its analogs on the viability of HaCaT human keratinocyte-derived cells, A549 non-small cell lung adenocarcinoma-derived cells, and PC3 human prostate cancer-derived cells are summarized in Table 4. Raniseptin-PL displayed high cytolytic activity against these cells (LC_50_ < 10 µM). The potencies of the [D4K] and [D4k] analogs were not significantly different from those of the native peptide but substitution of the Leu^18^ residue by L-lysine in the [L18K] and [D4K,L18K] analogs increased the LC_50_ values by between 2- and 4-fold (*p* < 0.05). Importantly, the presence of a D-lysine residue in the [L18k] and [D4k,L18k] analogs abolished cytolytic activity against the three cells lines at concentrations up to 100 µM. Similarly, the substitution Leu^18^ → D-Lys strongly attenuated hemolytic activity with the [D4k,L18k] analog producing no detectable hemolysis at 300 µM after a 1 h incubation at 37 °C.

## 3. Discussion

The therapeutic potential of frog skin HDPs as antimicrobial agents is limited by their often relatively low potency and rapid clearance from the circulation compared with non-peptidyl agents as well as their relatively high cytotoxicity against mammalian cells. Several strategies have been adopted to increase antimicrobial activity and stability concomitant with decreasing toxicity with a view to promoting their utility for systemic use [24,25]). The antimicrobial activities of cationic, α-helical peptides are determined by complex interactions between molecular charge, hydrophobicity, helicity and amphipathicity [26]. These are not independent variables as changes in one parameter may produce concomitant changes in another. In view of the fact that the bacterial cell membrane is associated with an overall negative charge compared with the zwitterionic plasma membrane of mammalian cells, one approach to increasing activity involves increasing cationicity while preserving amphipathicity by replacement of acidic (Asp and Glu) and neutral residues by L-Lys and/or L-Arg [27].

CD studies have shown that raniseptin-PL adopts an extended helical conformation in a range of membrane-mimetic environments with the substitutions Asp^4^ → Lys and Leu^18^ → Lys producing a small increase in % helicity. Interestingly, the AlphaFold models revealed small differences in the overall three-dimensional geometry of the helices, with a more pronounced bending in [L18K]raniseptin-PL compared with the native peptide (Supplementary Figure S4). This might promote insertion of the analog into the bacterial cell membrane and thus contribute to the small increases in potency seen in the case of some Gram-negative bacteria (Table 3). Taken together, these observations suggest that the L-Lys substitutions may subtly modulate α-helix stability and three-dimensional geometry rather than inducing a major change in secondary-structure organization. Although such geometrical differences could potentially contribute to variations in CD spectral intensity, the very similar [θ]_222_/[θ]_208_ ratios indicate that the overall α-helical organization is largely conserved among the three peptides in micellar environments.

Substitution of Asp^4^ by L-Lys produces an increase in cationicity from +5 to + 7 but this change resulted in only relatively minor increases in antimicrobial potency (approx. 2-fold) against both Gram-negative and Gram-positive bacteria and no significant change in the high cytotoxicity against human-derived cells. Increasing the molecular charge further to +8 by the double substitutions Asp^4^ → Lys and Leu^18^ → Lys did not increase potency against the Gram-negative strains and attenuated potency against *S. aureus*. Cytotoxicity of [D4K,L18K]raniseptin-PL against the human-derived cells was between 3- and 4-fold less than the corresponding values for the [D4K] analog but LC_50_ concentrations in the range 16.2–32.5 µM preclude systemic administration. This result is consistent with a previous study in which it was shown that increasing the molecular charge on the frog skin HDP pseudin-2 from +2 in the native peptide to +5 by appropriate amino acid substitutions increased activity against clinical isolates of antibiotic-resistant *E. coli* by 4-fold but further increases in cationicity up to a charge of +8 did not result in additional increases in antibacterial potency and, in this case, enhanced cytotoxic activity [28].

Previous studies involving the frog skin HDPs ascaphin-8 [29], alyteserin-2a [30], hymenochirin-1Pa [31], kassinaturin-1 [32] and pseudin-2 [28,33] showed that incorporating D-lysine residues at selected sites within the molecules in order to increase cationicity while preserving amphipathicity increased antibacterial potency to varying degrees but reduced cytotoxic activity against mammalian cells. The present study has demonstrated that substitution of Leu^18^ in the central region of the frog skin HDP raniseptin-PL by D-Lys has a differential effect on antimicrobial activity and on cytotoxic selectivity for bacteria and eukaryotic cells. The analogs [L18k]raniseptin-PL and [D4k,L18k]raniseptin-PL display potent antimicrobial activity (MIC ≤ 12.5 µg.mL^−1^) against the Gram-negative ESKAPE^+^ pathogens *A. baumannii* and *E. coli* and against antibiotic-resistant strains of *K. pneumoniae* and *P. aeruginosa* but reduced activity against Gram-positive *S. aureus* and *E. faecium.* Importantly, the cytotoxicity of the [L18k] and [D4k,L18k] analogs against non-neoplastic human-derived HaCaT keratinocytes and against A549 non-small cell lung adenocarcinoma cells and PC-3 prostate cancer cells was strongly attenuated (LC_50_ > 100 µM) compared with the naturally occurring peptide, thus fulfilling the therapeutic requirement for non-toxicity. Of particular note, the % hemolysis produced by incubation for 1 hr at 37 °C of 300 µM [L18k]raniseptin-PL with human red blood cells was only 4.6% and hemolysis was undetectable after the corresponding incubation with 300 µM [D4k,L18k]raniseptin-PL (Table 4).

Gram-positive bacteria are associated with a relatively rigid cell wall (20–80 nm thick) composed of cross-linked peptidoglycan layers incorporating negatively charged teichoic, teichuronic and lipoteichoic acid polymers that is absent in Gram-negative bacteria [34]. At this point in time the PEP-FOLD4 and AlphaFold servers cannot be used to predict the conformation of peptides containing D-amino acid residues, but the observation that Leu^18^ → D-Lys substitution strongly attenuates activity against Gram-positive bacteria invites speculation. Incorporation of a D-lysine residue into an antimicrobial peptide disrupts the α-helical conformation [35] such that passage across the outer cell wall is either impaired or prevented. The much thinner outer lipopolysaccharide membrane of Gram-negative bacteria is stabilized by Mg^2+^ and Ca^2+^ ions. The increase in cationicity produced by the Leu^18^ substitution facilitates displacement of these stabilizing ions, thereby facilitating passage across the membrane and allowing access to the periplasm [36]. This leads ultimately to loss of integrity of the inner phospholipid membrane and cell death.

Frog skin HDPs with high cytotoxicity against mammalian cells have received attention as potential anti-cancer agents, particularly in situations in which tumors have developed resistance to previously effective drugs [37]. Consistent with previous studies using MDA-MB-231 human breast adenocarcinoma cells, HT-29 human colorectal adenocarcinoma cells and human umbilical vein endothelial cells (HUVECs) [15], raniseptin-PL is strongly cytotoxic to A549 and PC3 human tumor cells (LC_50_ < 10 µM) (Table 4). The potencies of the [D4K] and [D4k] analogs were similar to those of the native peptide but their potential for development into therapeutically valuable anti-cancer agents is limited by the fact the peptides are not selective for tumor-derived cells. The LC_50_ values against non-neoplastic HaCaT cells are comparable to those determined for the tumor-derived cells.

A serious limitation to the use of frog skin HDPs as therapy for systemic infections is the rapid clearance of the peptides in the circulation. At this time the only frog skin peptide to have undergone extensive clinical trials is pexiganan, a lysine-substituted analog of magainin-2 first isolated from *Xenopus laevis* [38] that was designated for topical application to treat diabetic foot ulcers [39]. The compound ultimately failed to gain FDA approval and no frog skin peptide has been approved for systemic application. A number of strategies for increasing the half-life of biologically active peptides have evolved such as conjugation to fatty acids to facilitate binding to albumin, conjugation to polyethylene glycol, incorporation of unnatural amino acids, including D-amino acids, to inhibit the action of endopeptidases, chemical modification of NH_2_- and COOH-terminal amino acids to inhibit degradation by exopeptidases, and covalent chemical cross-linking to lock the peptide in a rigid α-helical conformation (“peptide stapling”) (reviewed in [25,40]). Further studies are warranted in which [L18k]raniseptin-PL and [D4k,L18k]raniseptin-PL are used as templates to develop long-acting analogs for use in the treatment of systemic infections caused by Gram-negative bacteria including ESKAPE^+^ pathogens. Future investigations will compare the metabolic clearance rates and half-lives of the [L18k] and [D4k,L18k] analogs in mice with those of raniseptin-2PL and investigate the efficacy of the peptides in promoting survival in appropriate animal models of bacterial infection.

## 4. Materials and Methods

### 4.1. Synthetic Peptides

Raniseptin-PL and its [D4K], [L18K], [D4K,L18K], [D4k], [L18k], and [D4k,L18k] analogs were supplied in crude form by Synpeptide Co., Ltd. (Shanghai, China), and purified to near homogeneity (>98% purity) by reversed-phase HPLC under the conditions previously described [15]. The identities of the peptides were confirmed by electrospray mass spectrometry. Full details are provided in the Supplementary Materials.

### 4.2. Circular Dichroism Studies

Circular dichroism spectra were recorded on a MOS-500 Circular Dichroism Spectrometer (BioLogic, Seyssinet-Pariset, France). Full details are provided in the Supplementary Materials. The spectra of raniseptin-PL and its analogs were recorded in 20 mM phosphate buffer (pH 7.0) under the following conditions: buffer alone, 50% (*v*/*v*) trifluoroethanol (TFE), 80% (*v*/*v*) methanol, 20 mM dodecylphosphocholine (DPC), and 20 mM sodium dodecyl sulfate (SDS). All solutions contained phosphate buffer at pH 7. Peptide secondary structure was estimated using the SELCON3 algorithm available through the ChiraKit platform [21,22]. Peptide α-helical content was also calculated by using the Forood formula, 100 × ([θ]_222_/max[θ]_222_) with max[θ]_222_ = −40,000 [1 − (2.5/*n*)], where n = number of amino acid residues [23]. The ratio [θ]_222_/[θ]_208_ was also calculated as an estimate of the degree of helicity.

### 4.3. Peptide Structure Prediction

The three-dimensional structures and α-helical propensity of raniseptin-PL and its analogs were predicted using two complementary computational approaches, the PEP-FOLD4 [16] and AlphaFold servers [17]. Mol* Viewer was used for visualization and analysis of structures [41]. Detailed descriptions of the prediction procedures and results obtained with both approaches are provided in the Supplementary Materials.

### 4.4. Antimicrobial Assays

Minimum Inhibitory Concentration (MIC) and Minimum Bactericidal Concentration (MBC) of raniseptin-PL and its analogs were determined against the following strains of ESKAPE^+^ bacterial pathogens: the Gram-negative aerobes *A. baumannii* ATCC 19606, *E. coli* ATCC 25922 and DSM 787, carbapenem-resistant *K. pneumoniae* (CRE) ATCC BAA 1705, *P. aeruginosa* PA01 WT ATCC 15692 and carbapenem-resistant *P. aeruginosa* PA01 DSM 50071 and the Gram-positive aerobes *E. faecalis* ATCC 29212 and vancomycin-resistant *E. faecalis* (VRE) MF06036, vancomycin-resistant *E. faecium* (VRE) NCTC 12201, *Staphylococcus epidermidis* ATCC 12228, *S. aureus* ATCC 25923 and methicillin-resistant *S. aureus* (MRSA) ATCC 43300. Activity against a strain of *Enterobacter* spp. was not investigated in this study. Assays were performed according to procedures mandated by CLSI M07 standards [42]. Ciprofloxacin (0.015–2.0 µg.mL^−1^) was used as a positive control. Full details are provided in the Supplementary Materials.

### 4.5. Cytotoxicity Assays

All cell lines were sourced from the American Type Culture Collection (ATCC). Cytotoxicities against the HaCaT human keratinocyte cell line (RRID: CVCL_0038), the A549 non-small cell lung adenocarcinoma cell line (RRID: CVCL_0023), and the PC-3 human prostate cancer cell line (RRID: CVCL_0035) were measured using a MTT (3-(4,5-dimethylthiazol-2-yl)-2,5-diphenyltetrazolium bromide) assay kit supplied by Thermo Fisher Scientific (Waltham, MA, USA) following the manufacturer’s recommended protocol. The effects of the peptides (0.78–100 μM) on cell viability were determined following a 24 h incubation at 37 °C. Full details are provided in the Supplementary Materials. The LC_50_ value was taken as the mean concentration of peptide producing 50% cell death in a minimum of three independent experiments.

Hemolytic activity of the peptides (37.5–300 µM) against human erythrocytes was determined as previously described [15]. Full details are provided in the Supplementary Materials. The LC_50_ value was taken as the mean concentration of peptide producing 50% hemolysis in three independent experiments.

### 4.6. Statistical Analysis

Data were compared using an unpaired Student’s *t* test with two-tailed *p* values and 95% confidence intervals. Values are considered to be significantly different if *p* < 0.05.

## 5. Conclusions

The study has shown that replacement of the L-leucine residue at position 18 in the frog skin HDP raniseptin-PL by D-lysine generates an analog with potent activity against Gram-negative bacteria including selected ESKAPE^+^ pathogens while concomitantly strongly attenuating cytotoxicity against eukaryotic cells. Consequently, [D-Lys^18^]raniseptin-PL and [D-Lys^4^,D-Lys^18^]raniseptin-PL represent templates for the design of “second-generation” long-acting therapeutic agents for systemic use in infections produced by such bacteria.

## Figures and Tables

**Figure 1:**
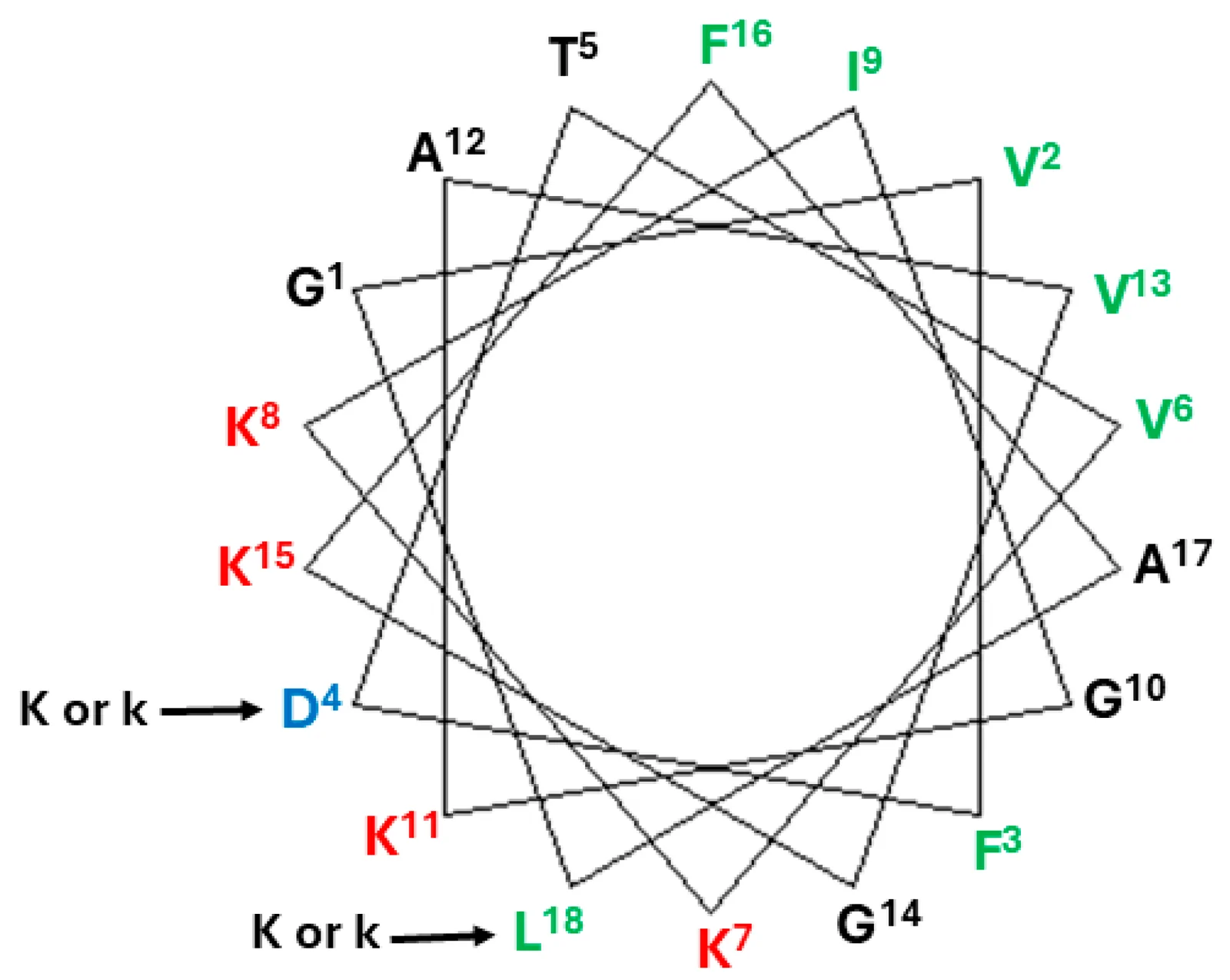
Schiffer–Edmundson wheel representation of raniseptin-PL illustrating the amphipathic helical character of the secondary structure. K = L-lysine, k = D-lysine. Basic residues are shown in red, acidic residues in blue and hydrophobic residues in green.

**Figure 2:**
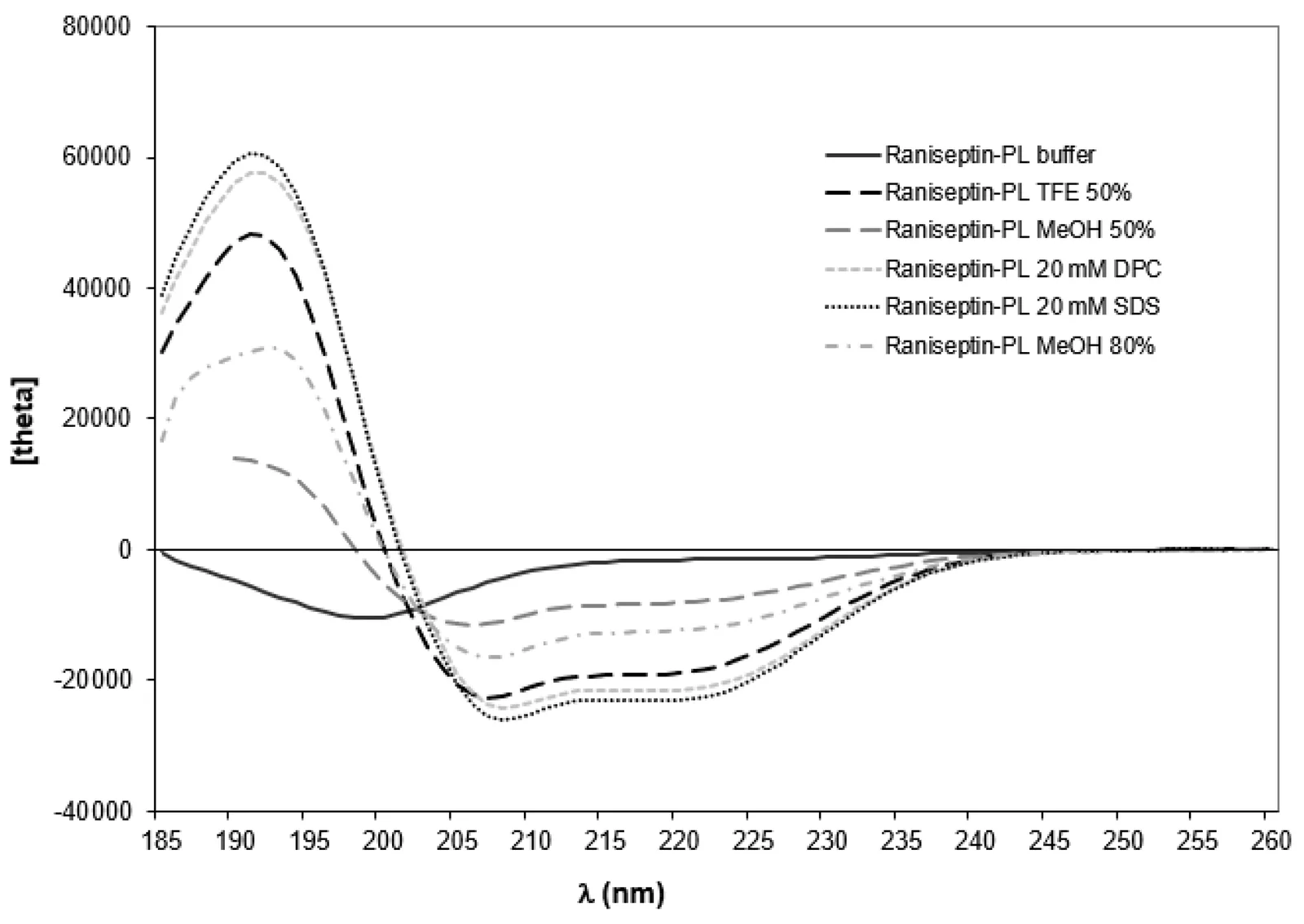
Circular dichroism spectra of raniseptin-PL recorded at room temperature at a peptide concentration of 0.2 mg.mL^−1^ in 20 mM phosphate buffer (pH 7) under the following conditions: buffer alone, 50% (*v*/*v*) trifluoroethanol (TFE), 20 mM dodecylphosphocholine (DPC), and 20 mM sodium dodecyl sulfate (SDS). Spectra in 80% (*v*/*v*) methanol were obtained in 10 mM phosphate buffer and 0.13 mg.mL^−1^ peptide due to restricted solubility in this solvent.

**Figure 3:**
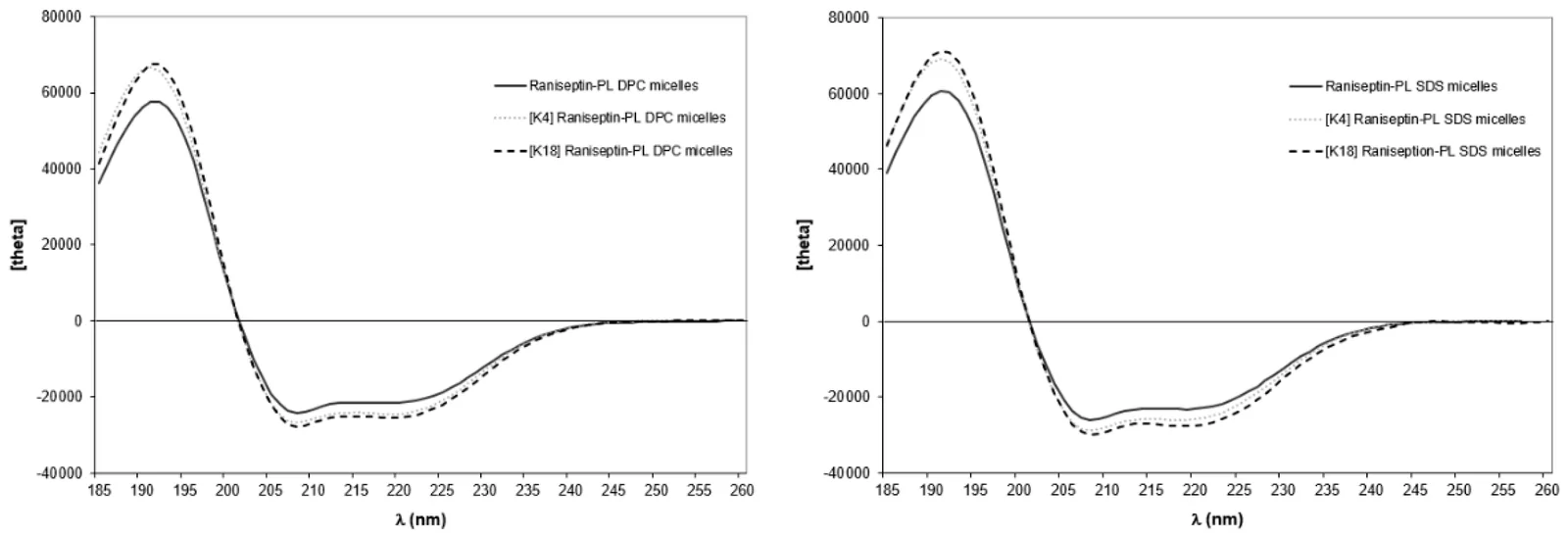
Circular dichroism spectra of raniseptin-PL, [D4K]raniseptin-PL, and [L18K]raniseptin-PL recorded at room temperature at a peptide concentration of 0.2 mg.mL^−1^ in 20 mM dodecylphosphocholine (DPC) (**left** panel) and 20 mM sodium dodecyl sulfate (SDS) (**right** panel). All solutions contained 20 mM phosphate buffer (pH 7).

**Table 1:** Primary structures and physicochemical properties of the ranaseptin-PL peptides used in this study.

Peptide	Primary Structure	Charge	H	µ
Raniseptin-PL	GVFDTVKKIGKAVGKFALGVAKNYLNS ^a^	+5	0.328	0.433
[D4K]	GVFKTVKKIGKAVGKFALGVAKNYLNS ^a^	+7	0.320	0.441
[L18K]	GVFDTVKKIGKAVGKFAKGVAKNYLNS ^a^	+6	0.228	0.502
[D4K,L18K]	GVFKTVKKIGKAVGKFAKGVAKNYLNS ^a^	+8	0.220	0.510
[D4k]	GVFkTVKKIGKAVGKFALGVAKNYLNS ^a^	+7	0.320	ND
[L18k]	GVFDTVKKIGKAVGKFAkGVAKNYLNS ^a^	+6	0.228	ND
[D4k,L18k]	GVFkTVKKIGKAVGKFAkGVAKNYLNS ^a^	+8	0.220	ND

Charge refers to molecular charge at pH 7.0. Hydrophobicity (H) was calculated using the hydrophobicity scale of Fauchère et al. [18] and hydrophobic moment (µ) [19]) using the HeliQuest webserver [20]. ^a^ denotes C-terminal α-amidation. ND: not determined. K = L-lysine, k = D-lysine.

**Table 2:** Prediction of secondary structure content of raniseptin-PL and its analogs from CD spectra. The values shown are %.

Peptide	Medium	Helix	β-Sheet	Turns	Random
Raniseptin-PL	Water	9 (4) [0.34]	30	15	44
	20 mM DPC	70 (58) [0.86]	1	8	23
	20 mM SDS	72 (62) [0.86]	0	8	23
	50%TFE	59 (50) [0.81]	4	10	27
	80% MeOH	45 (33) [0.73]	12	14	30
[D4K]	Water	10 (3) [0.26]	23	14	53
	20 mM DPC	74 (65) [0.88]	0	8	21
	20 mM SDS	77 (69) [0.86]	0	8	15
[L18K]	Water	8 (5) [0.36]	25	14	51
	20 mM DPC	75 (68) [0.89]	0	9	17
	20 mM SDS	75 (74) [0.89]	0	8	20

SELCON3 estimations [21] were obtained using the ChiraKit website [22]. Values in the ( ) parentheses show the % helicity calculated using the Forood formula [23] and values in the [ ] parentheses show the [θ]_222_/[θ]_208_ ratio. DPC: dodecylphosphocholine, SDS: sodium dodecyl sulfate, TFE: trifluoroethanol.

**Table 3:** Minimum Inhibitory Concentrations (µg.mL^−1^) for raniseptin-PL and its analogs against a range of clinically relevant Gram-positive and Gram-negative aerobic pathogens.

Pathogen	Raniseptin-PL	[D4K]	[L18K]	[D4K,L18K]	[D4k]	[L18k]	[D4k,L18k]
*E. coli* ATCC25922	12.5	6.25	6.25	12.5	3.13	3.13	6.25
*E. coli* DSM787	6.25 (6.25)	3.13 (3.13)	3.13 (3.13)	3.13 (3.13)	3.13 (3.13)	6.25 (6.25)	3.13 (6.25)
*S. aureus* ATCC 25923	12.5	6.25	100	100	50	>100	>100
*S. aureus* ATCC 43300	12.5 (12.5)	3.13 (3.13)	50 (100)	12.5 (25)	6.25 (6.25)	>100 (>100)	>100 (>100)
*E. faecium* NCTC 12201	50	50	25	100	12.5	100	>100
*S. epidermidis* ATCC 12238	3.13	3.13	3.13	3.13	3.13	50	50
*P. aeruginosa* ATCC 16692	6.25	3.13	3.13	3.13	3.13	12.5	6.25
*P. aeruginosa* DSM 50071	50 (100)	6.25 (12.5)	25 (25)	6.25 (6.25)	12.5 (25)	25 (50)	25 (25)
*A. baumannii* ATCC 19606	3.13	3.13	3.13	3.13	3.13	3.13	3.13
*E. faecalis* ATCC 29212	25	25	100	>100	50	>100	>100
*E. faecalis* MF06036	6.25 (12.5)	3.13 (3.13)	6.25 (12.5)	1.56 (3.13)	3.13 (6.25)	3.13 (6.25)	25 (25)
*K. pneumoniae* ATCC BAA1705	25 (25)	6.25 (12.5)	12.5 (12.5)	3.13 (6.25)	6.25 (12.5)	12.5 (12.5)	6.25 (6.25)

The values in parentheses show the Minimum Bactericidal Concentration (µg.mL^−1^).

**Table 4:** Cytotoxicities of raniseptin-PL and its analogs against human HaCaT keratinocyte cells, A549 lung adenocarcinoma cells, PC-3 prostate cancer cells and red blood cells (RBCs).

Cell	Rani-PL	[D4K]	[L18K]	[D4KL18K]	[D4k]	[18k]	[D4k,L18k]
HaCaT	8.7 ± 0.08	8.3 ± 0.08	20.8 ± 0.07	32.5 ± 0.05	3.5 ± 0.11	>100	>100
A549	4.8 ± 0.06	6.1 ± 0.06	18.0 ± 0.06	16.2 ± 0.06	7.1 ± 0.18	>100	>100
PC-3	6.6 ± 0.07	5.4 ± 0.06	15.2 ± 0.08	16.2 ± 0.05	6.9 ± 0.06	>100	>100
RBC	235 ± 25	245 ± 15	>300 (29)	>300 (33)	290 ± 14	>300 (4.6)	>300 (0.0)

Data show mean LC_50_ values (µM ± S.D.). The values in parentheses show % hemolysis at 300 µM.

## Data Availability

The original contributions presented in this study are included in the article/Supplementary Material. Further inquiries can be directed to the corresponding author.

## Supplementary Materials

The following supporting information can be downloaded at https://www.mdpi.com/article/10.3390/antibiotics15100944/s1.
