# Supplementary material for: Design of Potent, Non-Toxic Antimicrobial Agents Derived from the Frog Skin Peptide Raniseptin-PL Active Against Gram-Negative ESKAPE+ Pathogens
Source: Antibiotics (Basel). 2026 Sep 23;15(10):944. doi: 10.3390/antibiotics15100944 (PMC13700638; doi:10.3390/antibiotics15100944)
Supplement: Supplementary file 1 [file antibiotics-15-00944-s001.zip › antibiotics-4566442-supplementary.pdf]

Supplementary Material

Peptide synthesis

All peptides were purified to near homogeneity (> 98% purity), assessed by symmetrical peak shape and mass spectrometry, by reversed-phase HPLC on a (2.2 cm x 25 cm) Vydac 218TP1022 (C-18) column equilibrated with acetonitrile/water/ TFA (35.0/64.9.9.9/0.1, v/v/v) at a flow rate of 6 mL.min^{-1}. The concentration of acetonitrile was raised to 63% (v/v) over 60 min using a linear gradient. Absorbance was measured at 214 nm and the major peak in the chromatogram was collected manually. The identities of the peptides were confirmed by electrospray mass spectrometry.

Circular dichroism studies

Circular dichroism spectra were recorded on a MOS-500 Circular Dichroism Spectrometer (BioLogic, Seyssinet-Pariset, France). Data points were collected from 260 to 185 nm, with an integration time of 2 s per point and a step size of 1 nm, using a 1.0 mm path length rectangular quartz cell. Measurements were carried out at room temperature. Raniseptin-PL and its analogs were dissolved in water containing 40 mM phosphate buffer (pH 7.0) at a concentration of 1.0 mg.mL^{-1}. Each stock solution was used to prepare samples containing 25% or 50% (v/v) trifluoroethanol (TFE), 25% or 50% (v/v) methanol, 20 mM dodecylphosphocholine (DPC), or 20 mM sodium dodecyl sulfate (SDS), at a final peptide concentration of 0.20 mg·mL^{-1}. All these samples contained 20 mM phosphate buffer (pH 7.0). Additional samples containing 80% (v/v) TFE or 80% (v/v) methanol were prepared with 10 mM phosphate buffer (pH 7.0) and a lower peptide concentration of 0.13 mg.mL^{-1} because the peptides exhibited limited solubility in TFE and methanol when the organic solvent content exceeded 50% (v/v). At 80% (v/v) TFE, decreasing the peptide concentration to 0.13 mg.mL^{-1} and the phosphate buffer concentration to 10 mM did not restore the intensity of the CD spectra, which remained lower than that observed at 50% TFE. In contrast, under the 80% (v/v) methanol condition, the spectral intensity was recovered at a peptide concentration of 0.13 mg.mL^{-1}. Importantly, decreasing the peptide concentration to 0.13 mg·mL^{-1} in DPC and SDS micellar solutions had no significant effect on the normalized CD spectra, consistent with good peptide solubility under these conditions. Three scans were accumulated and averaged for each sample. All spectra were corrected by subtraction of the background obtained for each peptide-free solution. Circular dichroism measurements are reported as mean residue molar ellipticity ($[\theta]_{MRE}$ (deg.cm^2. dmol^{-1}).

Prediction of secondary structure with PEP-FOLD4

The propensity of raniseptin-PL and its analogs to adopt α-helical conformations was assessed using the online PEP-FOLD4 Peptide Structure Prediction Server (RPBS), with the TS4 option for 3D structure generation. For each peptide, the five best-ranked structural models generated by the server were retained for analysis. In addition to the three-dimensional models, the Predicted Local Structure Profile provided by PEP-FOLD4 was examined to assess residue-specific secondary-structure propensity along the peptide sequence. The predicted models were also visualized according to secondary-structure type, allowing regions with a strong propensity to adopt an α-helical conformation to be distinguished from less structured or locally destabilized regions.

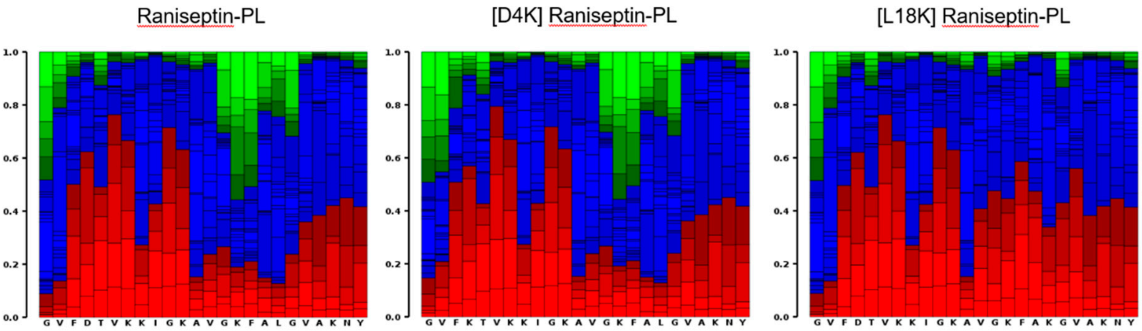

Figure S1. Predicted Local Structure Profile of raniseptin-PL and its analogs generated by PEP-FOLD4. The probabilities, at each position of the sequence are sorted from helical (red), coil (blue) to extended green.

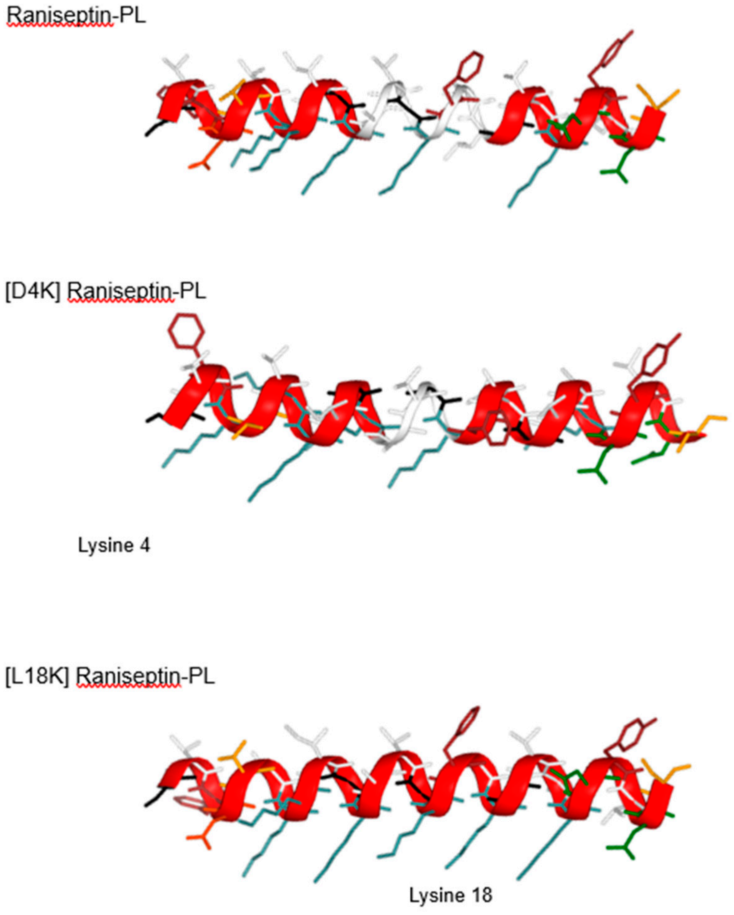

Figure S2. 3D structural representations of one of the five best models of raniseptin-PL and its analogs generated by PEP-FOLD 4. The peptide backbone is coloured according to the secondary structure prediction: helices are shown in red and loops or unstructured regions in gray. The predicted best models sometimes displayed non-linear topologies, with a two-helix architecture. Amino acid side chains are displayed according to their physicochemical properties.

Prediction of secondary structure with AlphaFold

The three-dimensional structures of raniseptin-PL and its analogs, [D4K]raniseptin-PL and [L18K]raniseptin-PL, were predicted using AlphaFold Server. For each peptide, the five structural models generated by AlphaFold Server were retained and analyzed independently. These five models were considered as an ensemble of structural predictions to assess the consistency of the predicted secondary structure and the variability in overall peptide geometry. The confidence of the predicted local structures was assessed using the predicted Local Distance Difference Test (pLDDT) score provided by AlphaFold. For all peptides and across all five predicted models, pLDDT scores were above 90 for residues 1–26, indicating very high confidence in the local structural predictions. The only exception was the C-terminal residue 27, for which the pLDDT score was lower but remained above 70 in all models.

The α-helical organization of each peptide was assessed from the backbone geometry of the five predicted structures. Backbone dihedral angles φ and ψ were calculated for each residue from the atomic coordinates of each model. The φ angle was defined by the C(i−1)–N(i)–Cα(i)–C(i) atoms, whereas the ψ angle was defined by the N(i)–Cα(i)–C(i)–N(i+1) atoms. Canonical α-helical backbone dihedral angles of $\varphi = -57°$ and $\psi = -47°$ were used as reference values. A tolerance of ±20° around each reference angle was applied as a stringent geometric criterion. Accordingly, a residue was classified as adopting a α-helix-compatible backbone conformation when both conditions were satisfied: $-77° \le \varphi \le -37°$ and $-67° \le \psi \le -27°$. This assignment was performed independently for each residue in each of the five AlphaFold-predicted models.

For each residue i, the α-helical frequency (Fα) across the five predicted models was calculated as

$F\alpha(i) = [N\alpha(i) / 5] \times 100$ where $N\alpha(i)$ is the number of models in which residue i simultaneously satisfied the φ and ψ criteria. As five models were analyzed for each peptide, the possible α-helical frequencies were 0, 20, 40, 60, 80, and 100%. A value of 100% indicates that the residue exhibited a α-helix-compatible backbone geometry in all five predicted structures. Given the consistently high pLDDT scores (>90 for residues 1–26) and the reproducibility of the α-helical backbone geometry across the five independently analyzed AlphaFold models, a high residue-specific α-helical frequency was considered indicative of a strong predicted propensity of the corresponding region to adopt an α-helical conformation.

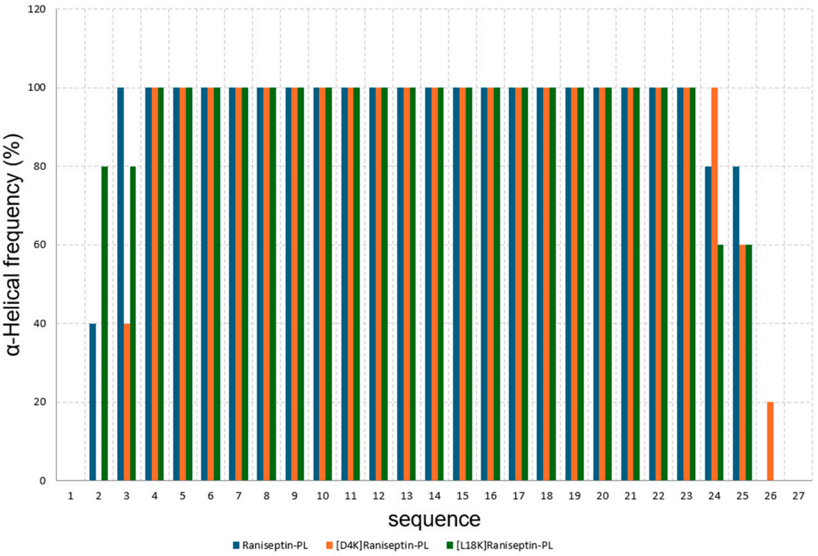

Figure S3. Residue-specific α-helical frequency of raniseptin-PL and its analogs predicted by AlphaFold. α-Helical frequencies are shown for raniseptin-PL, [D4K]raniseptin-PL, and [L18K]raniseptin-PL as a function of residue position.

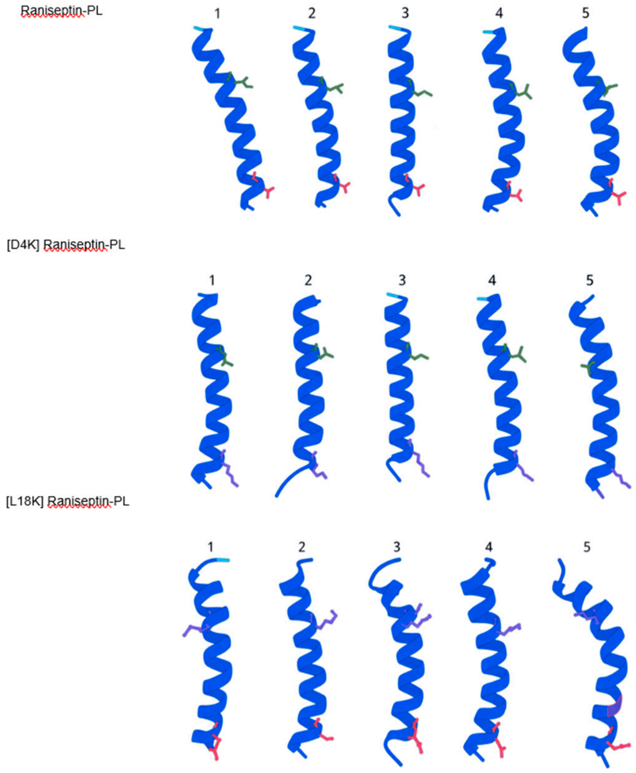

Figure S4. Models of Raniseptin-PL, [D4K] Raniseptin-PL, and [L18K] Raniseptin-PL generated by AlphaFold. Structural visualizations were prepared with the online Mol* viewer.

Determination of antibacterial activities

Assays were carried out at Ulster University, Coleraine U.K. and Sapienze University, Rome, Italy. The MICs for the aerobic bacteria were determined in duplicate using two separate inocula for each microbe in 96-well microtiter cell-culture plates by a standard double dilution method according to European Standard BS EN ISO 20776-1:2020 guidelines (equivalent to Clinical Laboratory and Standards Institute CLSI M07). To determine MBC, a 10 μL aliquot was taken from each well of the MIC assay mictrotiter plate, spotted onto tryptone soya agar plates, dried and incubated overnight at 37°C. MBC was determined as the first well contents (corresponding to lowest peptide concentration) which gave no visible colony growth in the spot after incubation.

Determination of cytotoxicities

Cytotoxicities were measured against human non-small cell lung adenocarcinoma cells A549, human prostate cancer cells PC-3 and human keratinocytes HaCaT. Cells were seeded in triplicates in a

96-well plate (6 x 103 cells/well), and were kept for 24 h at 37°C in a CO2 incubator. The effects of two-fold dilutions of the synthetic peptides (0.78 - 100 μM) on cell viability, after additional 24 h incubation at 37°C, were determined by measuring the formazan production by live cells using the reagent 3-(4,5-Dimethylthiazol-2-yl)-2,5-diphenyltetrazolium bromide (MTT) using an assay kit provided by Thermo Fisher Scientific, Waltham, MA, USA (Cat. # M6494). The formazan crystals were solubilized in dimethyl sulfoxide (100 μL/well) on a shaker for 15 min in the dark. Absorbance was measured at 570 nm using an ELISA plate reader Varioscan (Thermo Fisher Scientific). Four wells of medium alone were used as negative controls. The LC_{50} values (μM) were determined using GraphPad Prism 8.0.1 for Windows (GraphPad Software, Boston, MA, USA) as the mean concentration of peptide producing 50% cell death.

In the hemolysis assay, peptides in the concentration range (37.5 -300 μM) were incubated with washed human erythrocytes derived from outdated whole blood in Dulbecco's phosphate-buffered saline, pH 7.4 (100 μL) for 1 h at 37°C. After centrifugation (12 000 × *g* for 15 s), the absorbance of the supernatant at 450 nm was measured. A parallel incubation in the presence of 1% v/v Triton X-100 was carried out to determine the absorbance associated with 100% hemolysis. The LC_{50} value was taken as the mean concentration of peptide producing 50% hemolysis in three independent experiments.
